# 1-(2-Furo­yl)-3-(*o*-tol­yl)thio­urea

**DOI:** 10.1107/S1600536808020114

**Published:** 2008-07-05

**Authors:** Rodrigo S. Corrêa, O. Estévez-Hernández, J. Ellena, J. Duque

**Affiliations:** a Grupo de Cristalografía, Instituto de Física de São Carlos, Universidade de São Paulo, São Carlos, Brazil; b Department of Structure Analysis, Institute of Materials, University of Havana, Cuba

## Abstract

The title compound, C_13_H_12_N_2_O_2_S, was synthesized from furoyl isothio­cyanate and *o*-toluidine in dry acetone. The thio­urea group is in the thio­amide form. The central thio­urea fragment makes dihedral angles of 2.6 (1) and 22.4 (1)° with the ketofuran group and the benzene ring, respectively. The mol­ecular structure is stabilized by N—H⋯O hydrogen bonds. In the crystal structure, centrosymmetrically related mol­ecules are linked by a pair of N—H⋯S hydrogen bonds to form a dimer with an *R*_2_^2^(6) ring motif.

## Related literature

For general background, see: Aly *et al.* (2007 ▶); Koch (2001 ▶); Estévez-Hernández *et al.* (2007 ▶). For related structures, see: Theodoro *et al.* (2008 ▶); Duque *et al.* (2008 ▶). For the synthesis, see: Otazo-Sánchez *et al.* (2001 ▶).
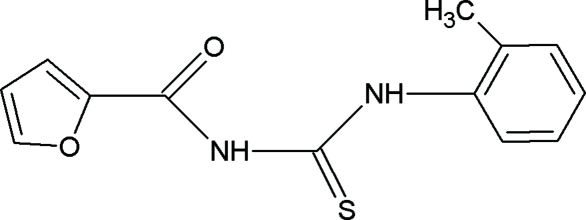


## Experimental

### 

#### Crystal data

C_13_H_12_N_2_O_2_S
*M* *_r_* = 260.31
Monoclinic, 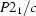

*a* = 6.0976 (1) Å
*b* = 16.6689 (6) Å
*c* = 13.1462 (4) Å
β = 108.765 (2)°
*V* = 1265.16 (6) Å^3^
*Z* = 4
Mo *K*α radiation
μ = 0.25 mm^−1^
*T* = 294 K
0.50 × 0.08 × 0.07 mm

#### Data collection

Nonius KappaCCD diffractometer
Absorption correction: Gaussian (Coppens *et al.*, 1965 ▶) *T*_min_ = 0.925, *T*_max_ = 0.983
8242 measured reflections
2408 independent reflections
1594 reflections with *I* > 2σ(*I*)
*R*_int_ = 0.048

#### Refinement

*R*[*F* ^2^ > 2σ(*F* ^2^)] = 0.046
*wR*(*F* ^2^) = 0.130
*S* = 1.02
2408 reflections
164 parameters
H-atom parameters constrained
Δρ_max_ = 0.26 e Å^−3^
Δρ_min_ = −0.30 e Å^−3^

### 

Data collection: *COLLECT* (Nonius, 2000 ▶); cell refinement: *SCALEPACK* (Otwinowski & Minor, 1997 ▶); data reduction: *DENZO* (Otwinowski & Minor, 1997 ▶) and *SCALEPACK*; program(s) used to solve structure: *SHELXS97* (Sheldrick, 2008 ▶); program(s) used to refine structure: *SHELXL97* (Sheldrick, 2008 ▶); molecular graphics: *ORTEP-3 for Windows* (Farrugia, 1997 ▶); software used to prepare material for publication: *WinGX* (Farrugia, 1999 ▶).

## Supplementary Material

Crystal structure: contains datablocks global, I. DOI: 10.1107/S1600536808020114/ci2623sup1.cif

Structure factors: contains datablocks I. DOI: 10.1107/S1600536808020114/ci2623Isup2.hkl

Additional supplementary materials:  crystallographic information; 3D view; checkCIF report

## Figures and Tables

**Table 1:** Hydrogen-bond geometry (Å, °)

*D*—H⋯*A*	*D*—H	H⋯*A*	*D*⋯*A*	*D*—H⋯*A*
N1—H1⋯O2	0.86	2.26	2.682 (3)	110
N1—H1⋯S1^i^	0.86	2.80	3.639 (2)	165
N2—H2⋯O1	0.86	1.92	2.649 (2)	141

Symmetry code: (i) 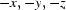.

**Fig. 1.**
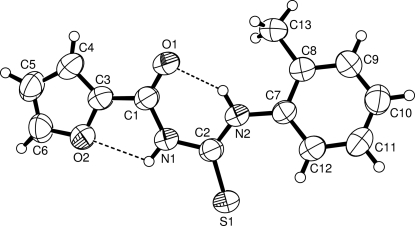
The molecular structure of the title compound, showing 50% probability displacement ellipsoids. Intramolecular hydrogen bonds are shown as dashed lines.

**Fig. 2.**
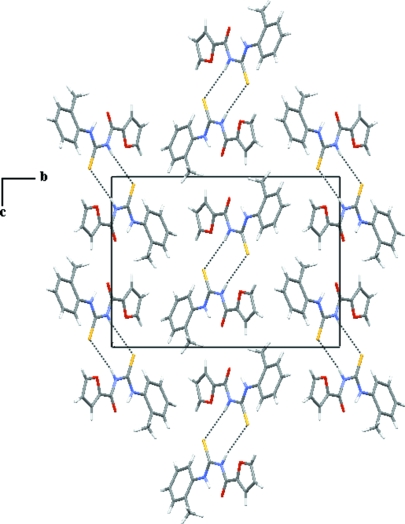
View of the crystal packing of the title compound. Intermolecular Hydrogen bonds are shown as dashed lines.

## supplementary crystallographic information

### Comment

Thiourea and its derivatives form a versatile family of ligands suitable to form complexes with ions of transition metals through the S atom (Aly *et al.*, 2007). Of analytical interest is the potential application of these compounds as ionophores or chemical modifiers in potenciometric and amperometric sensors (Estévez-Hernández *et al.*, 2007). The derived crystal structures help to understand the behaviour of these ligands as ionophores and also the complex formation with salts of the heavy metals. The title compound (Fig.1) is another example of our newly synthesized furoylthiourea derivatives.

The title compound crystallizes in the thioamide form. The bond lengths and angles are within the ranges observed for similar compounds (Koch, 2001). The C2—S1 [1.652 (2) Å] and C1—O1 [1.221 (2) Å] bonds both show the expected double-bond character. The short values of the C2—N1 [1.400 (3) Å], C2—N2 [1.330 (3) Å] and C1—N1 [1.377 (2) Å] bonds indicate partial double bond character. These results can be explained by the existence of resonance in this part of the molecule. The furan carbonyl group [O1/O2/C1/C3-C6] is nearly coplanar with the plane of the thiourea fragment [N1/N2/C2/S1, dihedral angle 2.6 (1)°], whereas the C7-C12 benzene ring is inclined by 22.4 (1)°. The *trans*-*cis* geometry in the thiourea group is stabilized by the N2—H2···O1 and N1—H1···O2 intramolecular hydrogen bonds (Fig.1 and Table 1). The crystal structure is stabilized by two intermolecular N1—H1···S1 hydrogen bonds (Fig.2 and Table 1) between centrosymmetrically related molecules forming dimers stacked along the [100] direction.

### Experimental

The title compound was synthesized according to a previous report (Otazo-Sánchez *et al.*, 2001), by converting furoyl chloride into furoyl isothiocyanate and then condensing with *o*-toluidine. The resulting solid product was crystallized from ethanol yielding X-ray quality single crystals (m.p 387–388 K).

### Refinement

H atoms were placed in calculated positions with N-H = 0.86 Å and C-H = 0.93 Å (aromatic) or 0.96 Å (methyl), and refined in riding model, with *U*_iso_(H) = 1.5*U*_eq_(C_methy_l) and 1.2U_eq_(N,C_aromatic_).

### Crystal data

**Table:**

C_13_H_12_N_2_O_2_S	*F*_000_ = 544
*M**_r_* = 260.31	*D*_x_ = 1.367 Mg m^−^^3^
Monoclinic, *P*2_1_/*c*	Mo *K*α radiation λ = 0.71073 Å
Hall symbol: -P 2ybc	Cell parameters from 20621 reflections
*a* = 6.0976 (1) Å	θ = 2.9–25.7º
*b* = 16.6689 (6) Å	µ = 0.25 mm^−^^1^
*c* = 13.1462 (4) Å	*T* = 294 K
β = 108.765 (2)º	Needle, colourless
*V* = 1265.16 (6) Å^3^	0.50 × 0.08 × 0.07 mm
*Z* = 4	

### Data collection

**Table:**

Nonius KappaCCD diffractometer	*R*_int_ = 0.048
ω scans	θ_max_ = 25.7º
Absorption correction: Gaussian(Coppens *et al.*, 1965)	θ_min_ = 2.9º
*T*_min_ = 0.925, *T*_max_ = 0.983	*h* = −6→7
8242 measured reflections	*k* = −19→20
2408 independent reflections	*l* = −16→16
1594 reflections with *I* > 2σ(*I*)	

### Refinement

**Table:**

Refinement on *F*^2^	H-atom parameters constrained
Least-squares matrix: full	*w* = 1/[σ^2^(*F*_o_^2^) + (0.0702*P*)^2^ + 0.1015*P*] where *P* = (*F*_o_^2^ + 2*F*_c_^2^)/3
*R*[*F*^2^ > 2σ(*F*^2^)] = 0.046	(Δ/σ)_max_ < 0.001
*wR*(*F*^2^) = 0.130	Δρ_max_ = 0.26 e Å^−^^3^
*S* = 1.03	Δρ_min_ = −0.30 e Å^−^^3^
2408 reflections	Extinction correction: none
164 parameters	

### Special details

**Table:**

Geometry. All e.s.d.'s (except the e.s.d. in the dihedral angle between two l.s. planes) are estimated using the full covariance matrix. The cell e.s.d.'s are taken into account individually in the estimation of e.s.d.'s in distances, angles and torsion angles; correlations between e.s.d.'s in cell parameters are only used when they are defined by crystal symmetry. An approximate (isotropic) treatment of cell e.s.d.'s is used for estimating e.s.d.'s involving l.s. planes.

### Fractional atomic coordinates and isotropic or equivalent isotropic displacement parameters (Å^2^)

**Table:**

	*x*	*y*	*z*	*U*_iso_*/*U*_eq_	
S1	0.22254 (12)	0.09680 (4)	0.04759 (5)	0.0800 (3)	
N2	0.4198 (3)	0.09229 (10)	0.26210 (13)	0.0561 (4)	
H2	0.408	0.0712	0.3198	0.067*	
O2	−0.2979 (3)	−0.06394 (9)	0.17467 (12)	0.0679 (4)	
O1	0.1899 (3)	0.01628 (10)	0.37313 (12)	0.0757 (5)	
N1	0.0880 (3)	0.01904 (10)	0.18967 (13)	0.0578 (5)	
H1	−0.0083	−0.0016	0.1328	0.069*	
C7	0.6105 (3)	0.14441 (12)	0.27881 (15)	0.0528 (5)	
C2	0.2552 (4)	0.07008 (12)	0.17262 (17)	0.0556 (5)	
C1	0.0568 (4)	−0.00258 (13)	0.28515 (17)	0.0587 (5)	
C12	0.7138 (4)	0.16151 (13)	0.20141 (17)	0.0631 (6)	
H12	0.6557	0.1389	0.1333	0.076*	
C3	−0.1472 (4)	−0.05056 (13)	0.27543 (17)	0.0594 (5)	
C8	0.7009 (4)	0.17683 (13)	0.38190 (17)	0.0609 (6)	
C9	0.8905 (4)	0.22716 (14)	0.40236 (19)	0.0728 (6)	
H9	0.9524	0.2494	0.4705	0.087*	
C4	−0.2232 (5)	−0.08806 (15)	0.3482 (2)	0.0774 (7)	
H4	−0.1528	−0.0881	0.4224	0.093*	
C10	0.9905 (4)	0.24531 (14)	0.3252 (2)	0.0737 (6)	
H10	1.1166	0.2799	0.3409	0.088*	
C11	0.9035 (4)	0.21225 (14)	0.2256 (2)	0.0703 (6)	
H11	0.9719	0.2238	0.1734	0.084*	
C13	0.5994 (4)	0.15856 (18)	0.46916 (17)	0.0835 (8)	
H13A	0.671	0.1921	0.5303	0.125*	
H13B	0.6264	0.1032	0.4896	0.125*	
H13C	0.4357	0.1687	0.4435	0.125*	
C6	−0.4688 (4)	−0.11084 (14)	0.1874 (2)	0.0769 (7)	
H6	−0.5959	−0.1291	0.1316	0.092*	
C5	−0.4295 (5)	−0.12709 (15)	0.2904 (2)	0.0831 (8)	
H5	−0.5214	−0.1585	0.319	0.1*	

### Atomic displacement parameters (Å^2^)

**Table:**

	*U*^11^	*U*^22^	*U*^33^	*U*^12^	*U*^13^	*U*^23^
S1	0.0983 (5)	0.0930 (5)	0.0458 (4)	−0.0272 (4)	0.0189 (3)	0.0060 (3)
N2	0.0625 (10)	0.0623 (10)	0.0453 (10)	−0.0051 (9)	0.0199 (9)	0.0046 (8)
O2	0.0709 (10)	0.0737 (10)	0.0649 (10)	−0.0096 (8)	0.0302 (8)	−0.0045 (7)
O1	0.0788 (10)	0.0981 (13)	0.0498 (9)	−0.0183 (9)	0.0201 (8)	0.0072 (8)
N1	0.0627 (10)	0.0649 (11)	0.0469 (9)	−0.0066 (9)	0.0192 (8)	0.0012 (8)
C7	0.0572 (11)	0.0511 (12)	0.0512 (12)	0.0023 (10)	0.0190 (9)	0.0062 (9)
C2	0.0664 (13)	0.0525 (12)	0.0508 (13)	−0.0005 (10)	0.0231 (11)	0.0011 (9)
C1	0.0652 (13)	0.0602 (13)	0.0542 (13)	0.0000 (11)	0.0240 (11)	0.0040 (10)
C12	0.0688 (13)	0.0661 (14)	0.0583 (13)	0.0001 (11)	0.0262 (11)	0.0032 (10)
C3	0.0642 (13)	0.0624 (14)	0.0553 (13)	0.0021 (11)	0.0245 (11)	0.0016 (10)
C8	0.0639 (13)	0.0641 (14)	0.0541 (13)	−0.0005 (11)	0.0184 (10)	0.0032 (10)
C9	0.0739 (14)	0.0746 (16)	0.0652 (15)	−0.0132 (12)	0.0158 (12)	−0.0037 (12)
C4	0.0832 (17)	0.0879 (17)	0.0700 (16)	−0.0091 (14)	0.0370 (14)	0.0092 (13)
C10	0.0684 (14)	0.0705 (16)	0.0810 (17)	−0.0085 (12)	0.0223 (13)	0.0066 (13)
C11	0.0675 (14)	0.0750 (15)	0.0765 (17)	0.0015 (12)	0.0343 (13)	0.0164 (13)
C13	0.0874 (17)	0.113 (2)	0.0507 (13)	−0.0239 (15)	0.0226 (12)	−0.0079 (13)
C6	0.0716 (15)	0.0789 (17)	0.0887 (19)	−0.0192 (13)	0.0376 (14)	−0.0161 (14)
C5	0.0880 (18)	0.0825 (18)	0.094 (2)	−0.0170 (14)	0.0506 (16)	0.0027 (15)

### Geometric parameters (Å, °)

**Table:**

S1—C2	1.652 (2)	C8—C9	1.383 (3)
N2—C2	1.330 (3)	C8—C13	1.500 (3)
N2—C7	1.411 (2)	C9—C10	1.375 (3)
N2—H2	0.86	C9—H9	0.93
O2—C6	1.355 (3)	C4—C5	1.403 (4)
O2—C3	1.366 (3)	C4—H4	0.93
O1—C1	1.221 (2)	C10—C11	1.361 (3)
N1—C1	1.377 (2)	C10—H10	0.93
N1—C2	1.400 (3)	C11—H11	0.93
N1—H1	0.86	C13—H13A	0.96
C7—C12	1.388 (3)	C13—H13B	0.96
C7—C8	1.397 (3)	C13—H13C	0.96
C1—C3	1.449 (3)	C6—C5	1.325 (4)
C12—C11	1.385 (3)	C6—H6	0.93
C12—H12	0.93	C5—H5	0.93
C3—C4	1.344 (3)		
			
C2—N2—C7	131.21 (17)	C10—C9—C8	122.1 (2)
C2—N2—H2	114.4	C10—C9—H9	118.9
C7—N2—H2	114.4	C8—C9—H9	118.9
C6—O2—C3	106.19 (18)	C3—C4—C5	106.5 (2)
C1—N1—C2	128.76 (18)	C3—C4—H4	126.7
C1—N1—H1	115.6	C5—C4—H4	126.7
C2—N1—H1	115.6	C11—C10—C9	119.5 (2)
C12—C7—C8	120.07 (19)	C11—C10—H10	120.2
C12—C7—N2	123.93 (19)	C9—C10—H10	120.2
C8—C7—N2	115.95 (17)	C10—C11—C12	120.4 (2)
N2—C2—N1	114.13 (17)	C10—C11—H11	119.8
N2—C2—S1	128.33 (16)	C12—C11—H11	119.8
N1—C2—S1	117.52 (16)	C8—C13—H13A	109.5
O1—C1—N1	123.5 (2)	C8—C13—H13B	109.5
O1—C1—C3	121.03 (19)	H13A—C13—H13B	109.5
N1—C1—C3	115.5 (2)	C8—C13—H13C	109.5
C11—C12—C7	120.0 (2)	H13A—C13—H13C	109.5
C11—C12—H12	120	H13B—C13—H13C	109.5
C7—C12—H12	120	C5—C6—O2	110.5 (2)
C4—C3—O2	109.6 (2)	C5—C6—H6	124.7
C4—C3—C1	132.6 (2)	O2—C6—H6	124.7
O2—C3—C1	117.80 (18)	C6—C5—C4	107.1 (2)
C9—C8—C7	117.88 (19)	C6—C5—H5	126.4
C9—C8—C13	120.0 (2)	C4—C5—H5	126.4
C7—C8—C13	122.15 (19)		
			
C2—N2—C7—C12	−24.6 (3)	N1—C1—C3—O2	−5.5 (3)
C2—N2—C7—C8	158.1 (2)	C12—C7—C8—C9	1.5 (3)
C7—N2—C2—N1	−177.53 (18)	N2—C7—C8—C9	178.90 (19)
C7—N2—C2—S1	0.8 (3)	C12—C7—C8—C13	−178.4 (2)
C1—N1—C2—N2	8.7 (3)	N2—C7—C8—C13	−1.0 (3)
C1—N1—C2—S1	−169.87 (17)	C7—C8—C9—C10	−0.3 (3)
C2—N1—C1—O1	−6.2 (4)	C13—C8—C9—C10	179.6 (2)
C2—N1—C1—C3	174.14 (18)	O2—C3—C4—C5	0.5 (3)
C8—C7—C12—C11	−1.5 (3)	C1—C3—C4—C5	−178.3 (2)
N2—C7—C12—C11	−178.73 (18)	C8—C9—C10—C11	−0.9 (4)
C6—O2—C3—C4	−0.1 (2)	C9—C10—C11—C12	0.9 (4)
C6—O2—C3—C1	178.91 (18)	C7—C12—C11—C10	0.3 (3)
O1—C1—C3—C4	−6.4 (4)	C3—O2—C6—C5	−0.3 (3)
N1—C1—C3—C4	173.3 (2)	O2—C6—C5—C4	0.6 (3)
O1—C1—C3—O2	174.84 (19)	C3—C4—C5—C6	−0.7 (3)

### Hydrogen-bond geometry (Å, °)

**Table:**

*D*—H···*A*	*D*—H	H···*A*	*D*···*A*	*D*—H···*A*
N1—H1···O2	0.86	2.26	2.682 (3)	110
N1—H1···S1^i^	0.86	2.80	3.639 (2)	165
N2—H2···O1	0.86	1.92	2.649 (2)	141

Symmetry codes: (i) −*x*, −*y*, −*z*.
